# VISTA and its ligands: the next generation of promising therapeutic targets in immunotherapy

**DOI:** 10.1186/s12935-023-03116-0

**Published:** 2023-11-07

**Authors:** Najibeh Shekari, Dariush Shanehbandi, Tohid Kazemi, Habib Zarredar, Behzad Baradaran, Seyed Amir Jalali

**Affiliations:** 1 Department of Immunology, School of Medicine, Shahid Beheshti University of Medical Sciences, Tehran, Iran; 2 Immunology Research Center, Tabriz University of Medical Sciences, Tabriz, Iran; 3 Department of Immunology, School of Medicine, Tabriz University of Medical Sciences, Tabriz, Iran; 4 Tuberculosis and Lung Disease Research Center, Tabriz University of Medical Sciences, Tabriz, Iran

**Keywords:** VISTA, VSIG-3, PSGL-1, Immune checkpoint inhibitor, Immunotherapy

## Abstract

V-domain immunoglobulin suppressor of T cell activation (VISTA) is a novel negative checkpoint receptor (NCR) primarily involved in maintaining immune tolerance. It has a role in the pathogenesis of autoimmune disorders and cancer and has shown promising results as a therapeutic target. However, there is still some ambiguity regarding the ligands of VISTA and their interactions with each other. While V-Set and Immunoglobulin domain containing 3 (VSIG-3) and P-selectin glycoprotein ligand-1(PSGL-1) have been extensively studied as ligands for VISTA, the others have received less attention. It seems that investigating VISTA ligands, reviewing their functions and roles, as well as outcomes related to their interactions, may allow an understanding of their full functionality and effects within the cell or the microenvironment. It could also help discover alternative approaches to target the VISTA pathway without causing related side effects. In this regard, we summarize current evidence about VISTA, its related ligands, their interactions and effects, as well as their preclinical and clinical targeting agents.

## Introduction

With the advent of immunotherapy, cancer treatment modalities have undergone a revolution heralding a new era of specialized treatments that are supposed to improve the chances of successful therapies. Indeed, immunotherapy aims to activate or suppress the immune system to boost anti-tumor responses or attenuate the specific adaptive immune response against self-antigens. Since lymphocytes are the most critical players in immune responses, activatory and inhibitory receptors within them are the center of attention during immunotherapy [1]. Immune checkpoint receptors (ICRs) regulate the balance between the immune system’s stimulatory and inhibitory pathways and help maintain immunosurveillance. With the introduction of the inhibitory ICRs, cytotoxic T-lymphocyte-associated protein 4 (CTLA-4), and programmed cell death protein 1 (PD-1) by Alison et al., more attention was paid to this field in cancer patients, and the initial immune checkpoint inhibitors (ICIs) showed remarkable therapeutic effects. In addition to CTLA-4 and PD-1, several ICRs have been identified over the years, and they are in various stages of clinical trials. Moreover, the number of cases receiving United States Food and Drug Administration (FDA) approval is on the rise. Their brilliant results in creating dramatic and long-term clinical outcomes, especially in refractory cancers such as non-small cell lung cancer (NSCLC) and melanoma, have raised hopes for cancer therapy [2–4].

Among the novel ICRs, VISTA, also known as B7-H5, PDCD-1 homolog (PD-1H), stress-induced secreted protein 1 (SISP1), death domain1alpha (DD1a), Gi24, and differentiation of embryonic stem cells 1 (Dies1) plays a central role in immune system functions, and its association with several human disorders, including autoimmune disease, inflammatory diseases, infection, and cancer was confirmed [5]. Although there has been considerable research regarding the function and role of VISTA and its potential therapeutic target, little information is available regarding the ligands of VISTA and their interactions. This article will provide a comprehensive review of VISTA, its related ligands' roles and functions, the impact of thier interactions, and the newest targeting agents for them to evaluate their potential as therapeutic targets in the future.

## VISTA structure

VISTA, as a type I transmembrane protein (55–65 kDa), is encoded by a gene called *VSIR* and is located on 10q22.1 within an intron of the CDH23 gene. VISTA protein structure (without signal peptide: 32 amino acids) contains 279-aa; 130-aa in the hyperglycosylated extracellular Ig-V domain, 33-aa in the stalk region, 20-aa in the transmembrane domain, and 96-aa in the cytoplasmic domain [6].

The extracellular domain (ECD) of VISTA adopts a canonical β-sandwich formation with the front face representing H-, A-, G-, F-, C-, and C′ β-strands, while the back face represents A′-, B-, E-, D-, and C′′ β-strands. Moreover, three disulfide bonds exist between the strands of B-F (Cys^22^–Cys^114^), A′-H (Cys^12^–Cys^146^), and CC′ loop-F (Cys^51^–Cys^113^) as a result of the presence of six cysteines. The ECD of VISTA, mainly the C–C′ loop, contains a considerable number of histidine (H) residues with a positive charge. These residues are responsible for forming pH-dependent binding sites, which enhance the binding of VISTA to its ligands in acidic environments such as tumor microenvironment (TME) [5, 7]. The cytoplasmic region of VISTA does not contain tyrosine-based signaling motifs, including immunoreceptor tyrosine-based activation motif (ITAM), immunoreceptor tyrosine-based inhibitory motif (ITIM), and immunoreceptor tyrosine-based switch motif (ITSM). Additionally, the presence of proline-rich motifs, including three C-terminal Src homology domain 3 (SH3) binding motifs (PxxP) and a Src homology domain 2 (SH2) binding motif (YxxQ), as well as specific sequences for phosphokinase C (PKC) and casein kinase 2 (CK2) binding sites enable VISTA to alter some cellular functions within the cell and act both as a receptor and a ligand. The cytoplasmic region of VISTA, which is very important in terms of intracellular functions, is similar to members of the CD28 family, such as PD-1 and CTLA-4. Then, VISTA seems to share common functional properties with the CD28 family members [6, 7].

Phylogenetic analysis showed that VISTA has a highly conserved sequence, especially between humans and mice with 76% similarity. In light of the sequence similarity between VISTA and the other members of the B7 family, especially in the ECD, it has been classified as a member of this group. Analysis of the Ig-V domain within ECD showed that the highest homology rate between VISTA and B7 family members belongs to programmed death-ligand 1 (PD-L1), where 23% of the sequences are identical. Nevertheless, VISTA still exhibits some unique characteristics that make it stand unique from other members of the B7 family: (1) chromosomal locus in which *VSIR* gene is located apart from others, (2) conformational differences in the ECD domain; containing ten β-strands compared to nine in the B7 family fold, having an extra helix sequence (FQDL) and unique C–C′ unstructured loop of 21 residues, 3) Two extra disulfide bonds in IgV-like domain with unique cysteine residues (Cys44, Cys83, Cys144, and Cys177) that are absent in others, 4) While B7 family members have both IgC and IgV-like domains, VISTA lacks an IgC-like domain and exhibits extremely large IgV-like, and 5) lacking any ITAM, ITIM, ITSM motifs, which are existed in other B7 family members [5, 7].

## VISTA expression

At a steady state, VISTA is expressed in a wide range of human tissues, particularly hematopoietic compartments and tissues containing infiltrating leukocytes, e.g., bone marrow (BM), thymus, spleen, and lymph node [8, 9]. Evidence also indicates the presence of VISTA in secretory form [10, 11].

In terms of hematopoietic tissues, peripheral blood mononuclear cells (PBMCs), mainly myeloid lineages, including monocytes, myeloid dendritic cells (DCs), macrophages, neutrophils, and basophils have the highest expression level of VISTA [8, 9]. Concerning lymphoid cells with lower expression of VISTA than myeloid cells, while subsets of CD4 + T cells, exceedingly naïve CD4 + T cells, and forkhead box P3 (FoxP3 +) regulatory T cells (Tregs) exhibit higher levels of VISTA, CD8 + T cells, plasma cells, lymphoid DCs, CD56^low^ natural killer (NK) cells, and thymocytes express it at lower expression levels. However, its expression level on CD19 + B cells and CD56^high^ NK cells has not yet been observed. Regarding the expression of VISTA in mice, it has almost the same expression pattern as humans, mainly confined to hematopoietic tissues [8, 9].

Activation of immune cells could increase the expression level of VISTA depending on each cell type [6]. Nevertheless, this upregulation in CD4 + and CD8 + T cells decreases over time [5]. Hu et al. showed that activated CD4 + T cells showed higher expression levels of VISTA than activated CD8 + T cells; however, the intensity of increased expression was higher in activated CD8 + T cells than in activated CD4 + T cells [12]. It should be noted that experimental studies on monocytes also showed that VISTA expression gradually decreases after activation over time (in vitro after 24 h and in vivo after a few days) [6, 8, 13]. Yoon and colleagues showed upregulation of VISTA in apoptotic cells following DNA damage as a downstream target of p53 [14], and they suggested that surface expression of VISTA could be induced following apoptosis.

**Fig. 1:**
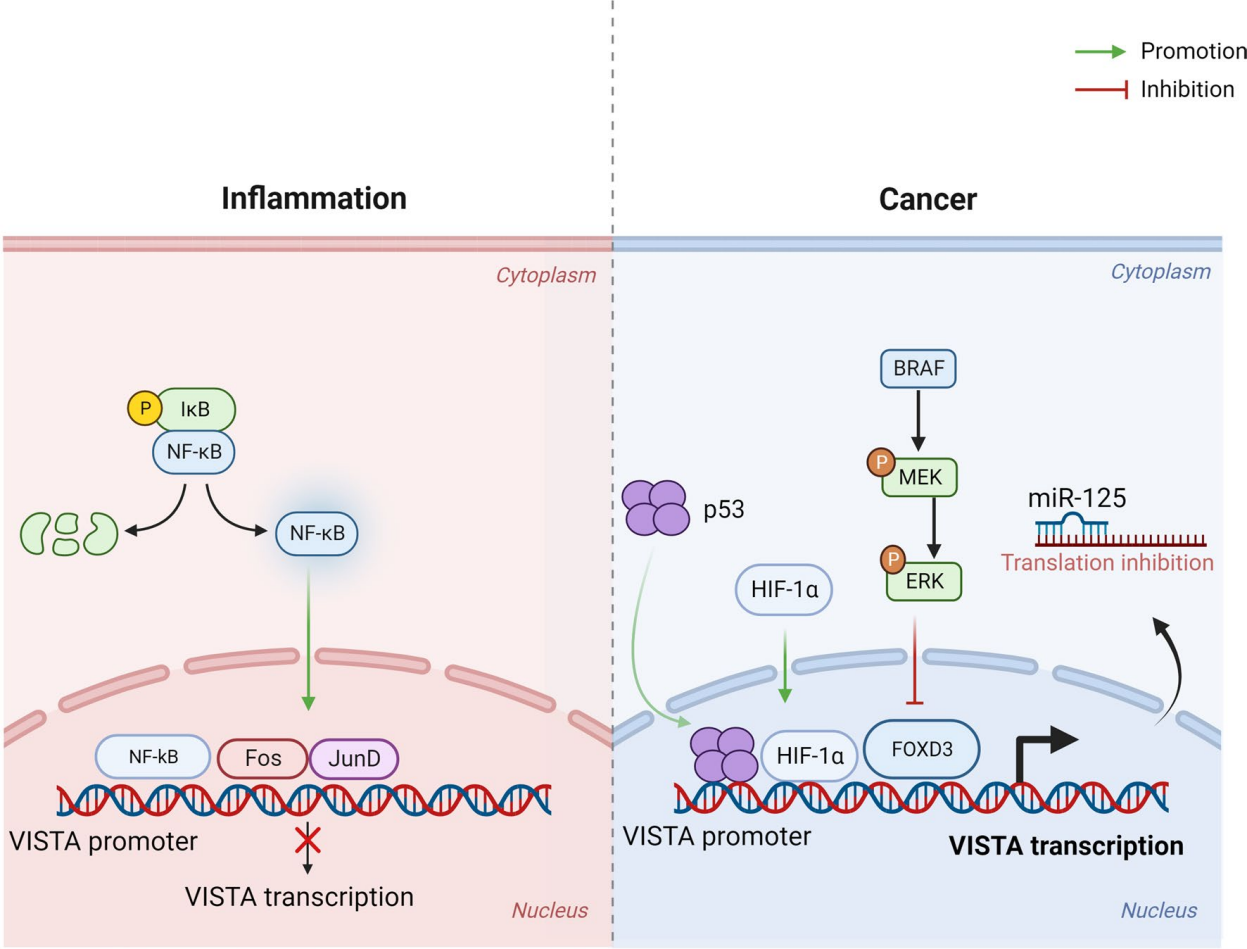
VISTA gene regulation in inflammation and cancer. Under inflammatory conditions, transcription factors, including NF-κB, Fos, and JunD attach to the VISTA promoter and suppress its transcription and expression. In tumor cells, increased p53 and HIF-1α bind to the promoter of VISTA and induce its expression. Additionally, BRAF neutralizes the FOXD3 inhibitory effect on VISTA expression by blocking the binding of FOXD3 to the VISTA promoter. Moreover, miR-125 has the ability to suppress VISTA translation. *VISTA* (V-domain immunoglobulin suppressor of T cell activation), *NF-κB* (nuclear factor kappa B), *HIF-1α* (hypoxia-inducible factor 1-alpha), *Forkhead box D3 (FOXD3)*, *miR-125* (microRNA-125). ***Created with BioRender.com***

Despite VISTA's expression pattern under various conditions, its regulatory network has not been adequately explored. In terms of transcriptional regulation, VISTA’s promoter is known to be bound by several transcription factors (TFs), including Fos, JunD, and nuclear factor kappa B (NF-κB). VISTA’s expression is reduced during inflammation periods in which Fos, JunD, and NF-κB are elevated [15, 16]. Other TFs that have an up-regulatory effect on VISTA expression, especially in the TME, are hypoxia-inducible factor 1-alpha (HIF-1α) and p53 [14, 17]. Additionally, Rosenbaum et al. showed the regulatory impact of the BRAF/Forkhead box D3 (FOXD3) pathway on VISTA expression where induced PD-L1 expression and increased Tregs in the TME [18]. Regarding the post-transcriptional level, evidence showed that microRNA (miRNA)-125a binding [19] to the mRNA of VISTA led to decreased VISTA expression within tumor cells. Furthermore, lipopolysaccharide (LPS) induced deacetylation of H3K27 (upstream of the VISTA gene) reduced VISTA expression and declared VISTA regulation at the epigenetic level [20]. A summary of VISTA gene regulation in inflammatory and cancerous conditions can be found in Fig. 1.

## VISTA’s functions in health and disease

VISTA plays a key role in regulating immune system responses and maintaining its homeostasis. Indeed, any changes in the homeostasis of the immune system (e.g., inflammation, autoimmunity, and cancer) make a change in the expression of VISTA within the immune cells, and any change in the expression level of VISTA could disrupt the homeostasis of the immune system and lead to autoimmune or inflammatory disorders [21]. As to which one happens first, there is not enough information available. In the following section, we will discuss the functions of VISTA and diseases affected by its expression and function.

As a receptor, VISTA’s inhibitory role on T cell functions was identified for the first time in a study on experimental autoimmune encephalomyelitis (EAE) as a mouse model for multiple sclerosis (MS), where its blockade led to the activation of T cell-mediated immunity and loss of peripheral tolerance [13]. The presence of VISTA on naïve CD4 + T cells and γδ-T cells prevents the activation of T cells in the absence of foreign antigens, which justifies the reduction in VISTA expression level in autoimmune disorders [22]. Moreover, an experimental conditional knockout (CKO) of VISTA in naïve T cells increased the number of T-bet^hi^ CD44^hi^ CD4 + T cells, indicating that VISTA is essential for T cell differentiation [16]. Regarding myeloid lineage, VISTA has inhibitory effects on macrophages and neutrophils functions, where anti-VISTA agonist antibodies (e.g., VISTA-COMP) induced immunoregulatory effects and decreased production of pro-inflammatory cytokines and chemokines in macrophages and neutrophils activated with LPS [23, 24]. Further studies confirmed that VISTA has a regulatory impact on chemokines expression and their receptors in myeloid cells: C5aR1 (monocytes and macrophages), CCR2 and CX3CR1 (monocytes), CD14 and CD16 (monocytes), CCL2, CCL3, CCL4, and CCL5 (macrophages), and CXCR2 (neutrophils) [25, 26]. Broughton et al. [25] showed that VISTA deficiency in macrophages reduced the presence of CCR2 and CCR5 on the cell surface in a manner that is not transcriptionally controlled (did not affect their mRNA levels). They further showed that this reduction led to the accumulation of CCL2 and CCL3 chemokines in culture supernatants, ultimately leading to a disturbance in the chemotaxis of macrophages and exacerbation of inflammation. However, the molecular mechanism involved was not identified, and their proposed hypothesis was the existence of a conserved motif in chemokine receptors that is recognized by VISTA-stimulated signaling proteins. VISTA also plays a role in the fate of myeloid cells. Usage of anti-VISTA agonist monoclonal antibody (mAb) following LPS stimulation showed that macrophages adopted a suppressive phenotype, a condition characterized by reducing pro-inflammatory agents (interleukin (IL)-12p40, tumor necrosis factor (TNF)-α, IL-6, and C-X-C motif chemokine (CXCL)-2 and increasing anti-inflammatory cytokines (IL-10), compared to the control group. Indeed, alterations in some TFs and signaling pathways made macrophages change their functional profile [23, 27]. Based on these findings, the relationship between VISTA and chemokine/chemokine receptors could influence myeloid cells fate in two different ways. One would be through a non-transcriptionally controlled mechanism, while the other involves altering the expression of chemokine genes. VISTA has the ability to control DCs and myeloid-derived suppressor cells (MDSCs)-mediated inflammation by regulating their Toll-like receptors (TLRs)/myeloid differentiation primary response 88 (MyD88) pathway [27]. Moreover, it contributes to monocytes and macrophages’ phagocytosis of apoptotic cells, debris (efferocytosis), and even myelin uptake in the central nervous system (CNS) [14, 28]. Furthermore, VISTA influences professional antigen-presenting cells (APCs) to suppress the antigen-presentation process [29].

As a ligand, it has been shown that using immobilized ECD of VISTA protein suppressed T cells in several activities, including signaling molecules, activation markers, cytokine production, and proliferation [5]. Furthermore, ECD of VISTA protein (MH5A agonist mAb) along with exogenous transforming growth factor beta (TGF-β) induced Foxp3 + adaptive Tregs differentiation [30]. In APCs, overexpression of VISTA within A20 cells (B lymphoma cells) or BM-DCs (BM-derived DCs) suppressed co-cultured transgenic CD4 + T cells (DO11.10) proliferation and cytokine production. VISTA protein on tumor cells was observed to overcome vaccine-induced anti-tumor immunity and promote tumor growth in the MCA105 fibrosarcoma model [13]. Therefore, it is very likely that VISTA inhibits the activity of T cells through interaction with a T cell co-inhibitory receptor. It is unclear whether VISTA utilizes different signaling pathways or molecular mechanisms in its two different functional states, so further research is required to answer this question.

Apart from VISTA's role in the immune system, it has been demonstrated that VISTA plays a role in the differentiation of mouse embryonic stem cells [31, 32] and the epithelial-to-mesenchymal-transition (EMT) process in some cancer cell lines [19]. It also has a regulatory effect on adipocyte production [33]. Given that they are not the focus of our discussion, we will not discuss them in further detail.

### Autoimmune and inflammatory disorders

VISTA's prominent roles under normal physiological conditions are mainly related to its expression within T and myeloid cells, especially naïve T cells, which eventually suppress their activation and maintain peripheral tolerance. Many types of autoimmune disorders appear to be caused by a disruption in VISTA's negative regulatory effects on the immune system responses, including suppressing the activity of T cells, DCs, macrophages, and γδ-T cells [21, 22].

Lupus is an autoimmune disease in which the immune system combats itself, causing complications in various organs. According to studies, there is a relation between VISTA expression and the development of lupus. Mice susceptible to lupus (Sle1.Sle3) showed a reduction in the expression level of VISTA on activated T cells and inflammatory monocytes in comparison to the control group [wild-type (WT)] mice, in which activation of T and myeloid cells and production of pro-inflammatory cytokines aggravated the progression of the disease [34]. In another study by Han et al., following VISTA deficiency in mice (VISTA KO), the number of immune cells increased, and inflammation intensified. However, they found that VISTA was increased within the immune cells of human discoid lupus erythematosus (DLE) biopsies, circulating immune cells of human systemic lupus erythematosus (SLE), and cutaneous lupus-like lesions of MRL/lpr mice as lupus-prone mice. Using VISTA agonist mAb (MH5A) in MRL/lpr, the number of activated immune cells and the level of pro-inflammatory cytokines decreased and made the inflammation subsided [35]. Additional studies confirmed that using VISTA-blocking mAb increased the number of inflammatory cells like activated T and myeloid cells and improved lupus progression in mice [36]. Therefore, it seems that the expression of VISTA in lupus appears to have two patterns; its expression is reduced in one type, whereas in the other type, its expression is increased, but its inhibitory activity is impaired. Thus, more research is needed in order to determine the role played by VISTA in the development of lupus.

MS is an autoimmune disease that occurs when the immune system attacks antigens located in the CNS. Wang et al. [13] reported an increase in the infiltration of interferon (IFN)-γ + and IL17A + producing CD4 + T cells within the CNS following VISTA blocking antibody (13F3), which indicated an increased susceptibility to EAE. Borggrewe et al. showed that in EAE mice, the expression of VISTA decreased in microglia cells. Nevertheless, they found that while inflammatory human MS lesions have more expression of VISTA, MS lesions without inflammation showed reduced expression. The differences in VISTA expression observed between human and mouse MS samples were attributed to the high heterogeneity of MS tissues and the difference in the rate of inflammation present in MS between the two species. They also suggested that the increased expression of VISTA in inflammatory MS tissues may be related to the presence of other immune cells and CNS resident cell types [28]. A study by Derakhshani et al. [37] on the PBMCs of relapsing–remitting multiple sclerosis (RRMS) patients revealed a reduction in VISTA expression, especially in different types of monocytes, compared to normal cases. They also showed that immunomodulating agents remarkably increased VISTA expression in these patients. As a result of these findings, it is recommended that when studying VISTA roles in MS, considerations should be taken into account for the heterogeneity and difference between mouse and human MS tissues, as well as the degree of inflammation and the type of cells studied.

In rheumatoid arthritis (RA) as an inflammatory and chronic systemic autoimmune disorder, VISTA in the synovium was involved in response to immune complexes and C5a in monocytes and macrophages [26]. The most important occurrence in psoriasis as a Th17-biased chronic skin inflammatory autoimmune disorder is inflammation caused by over-activated immune cells [38]. VISTA deficiency in DCs suppressed its regulatory effects on the TLR7 pathway and intensified inflammation via the IL-23/IL-17 mediated inflammatory axis in the psoriasis model induced by imiquimod (IMQ) [39]. The immunosuppressive effect of VISTA has also been reported in allergic skin inflammation, in which using MIH63 as anti-VISTA mAb enhanced ear swelling in the murine model [40].

Evidence shows that VISTA regulates T helper 2 (Th2)-mediated immune responses and related events, such as allergy reactions and disorders [41]. Even though recent studies indicate signs of autoimmune disease in asthma, it is still considered a non-autoimmune disorder. In particular, asthmatic patients' responses to immunosuppressive drugs indirectly support the autoimmune hypothesis [42]. VISTA deficiency in a mouse model of asthma promoted pulmonary eosinophilia and increased levels of Th2 and pro-inflammatory cytokines, which implies the regulatory role of VISTA in responses to allergens [41]. While VISTA agonist mAb (4C11) decreased asthma severity and lung inflammation [41], VISTA antagonist mAb (MIH63) increased Th2 cytokine levels and antibody production [43]. Moreover, Liu et al. [41] showed no significant differences in CD4 + and CD8 + T cells between VISTA KO and WT mice. However, the percentage of pulmonary and systemic CD4 + Foxp3 + Tregs decreased significantly, suggesting a possible role of Tregs in suppressing VISTA-mediated airway inflammation.

Using VISTA agonist mAb in a murine model of acute hepatitis suppressed inflammation primarily by suppressing the CD4 + T cell response [44]. However, in the context of HIV infection, there is an apparent increase in VISTA expression within human PBMC CD14 + monocytes following specific TLR stimulation (TLR3 and TLR5) and secretion of IL-10 and IFN-γ [8]. Also, in a study conducted on a murine model of AIDS (MAIDS), VISTA expressed within MDSC was found to suppress the proliferation of B cells [45]. Therefore, increasing VISTA expression in chronic inflammation such as HIV may represent an attempt to re-establish homeostasis following inflammation. As in bacterial infection, VISTA inhibition increased pro-inflammatory cytokines via the TLR/MyD88 pathway [27]. Regarding graft-versus-host disease (GVHD) as an inflammatory condition, VISTA expression exclusively in T cells is essential in modulating T cell responses [30]. Because pregnancy is both an anti-inflammatory and pro-inflammatory state, VISTA levels are increased during the first trimester to maintain allo-fetal tolerance, as indicated by studies in which VISTA and other members of the B7 family were abundantly present within the placenta during the pregnancy [9, 46].

### Cancer

As a result of disturbed immune homeostasis, the expression of ICs is altered within TME. Indeed, in order to escape anti-tumoral immune responses, TME conditions such as cytokines, hypoxia, and tumor-derived exosomes induce the expression of ICs, which eventually cause functional unresponsiveness and suppression of tumor-infiltrating lymphocytes (TILs) responses [47–49].

VISTA was initially shown to be present in TILs only [9, 50], but in later studies, it was found to be expressed in tumor cells [51–54], particularly in a cytoplasmic pattern and in a limited amount [55]. This is in contrast to the expression pattern of PD-L1, which has a broad expression, especially in hot tumors [50]. Several studies have examined the expression of VISTA genes or proteins in human cancers of different types in the way that they found a higher level of VISTA expression in tumor-infiltrating myeloid cells and -Tregs than in peripheral lymph nodes [21]. Boger et al. [56] found that TILs (> 80%) expressed higher levels of VISTA protein than tumor cells (< 10%) in gastric cancer (GC) tumor tissues. Furthermore, they showed a correlation between VISTA protein level and Epstein-Barr virus infection, tumor localization, Lauren classification, KRAS- and PIK3CA-mutational status, and PD-L1 expression. Conversely, in GC and gastrointestinal stroma tumor samples, Oliveira et al. [19] detected significantly reduced expression of VISTA compared to normal mucosa samples. However, VISTA protein expression did not demonstrate the same decrease in expression observed at the VISTA mRNA level. Furthermore, they mentioned the effects of genetics and epigenetics factors contributing to this reduction and recommended further investigation. It was shown that colorectal cancer patients have a very high expression of VISTA compared to normal and paratumor tissues [57]. Lei et al. [58] showed that the expression of VISTA was significantly elevated in liver cancer tissues, and inhibiting the VISTA signaling pathway reduced tumor cell growth and increased survival in mice with liver cancer. Zhang et al. [52] showed that the VISTA protein in tumor tissues of hepatocellular carcinoma and TILs is almost the same. Moreover, VISTA + /CD8 + patients showed better overall survival [52]. In a study by Kondo et al. [59], an increased expression level of VISTA in tumor tissues of human oral squamous cell carcinoma (OSCC) was reported, especially on CD11b + TILs. They found that blocking VISTA enabled CD8 + T cells to convert into functional effector cells. Therefore, it could be said that the overexpression of VISTA in TME can have different effects depending on the type of cancer. Although VISTA inhibits anti-tumor responses in TME, in some cases, an increase in the expression of VISTA is interpreted as a favorable prognosis and is associated with a higher survival rate [52, 60–62]. So, it is essential to conduct more research about how VISTA works concerning the type of cancer.

While initial findings indicated that VISTA cooperates with classical ICs (e.g., PD-1, PD-L1, and CTLA-4) to inhibit immune responses, later investigations revealed that VISTA by itself enhances immunosuppressive conditions via binding to its related ligands in TME. As Liu et al. showed, VISTA plays a non-redundant role in T cell activation, distinct from PD-1/PD-L1 [63, 64]. Deficiency or inhibition of VISTA in several cancers showed that the presence of myeloid cells with pro-inflammatory phenotype increased and further raised T-cell infiltration [25, 50]. Additionally, it has been shown that targeting VISTA promotes the efficacy of adoptive chimeric antigen receptor (CAR)-T cell immunotherapy in cancer [12]. Targeting VISTA on APCs within TME also eliminated VISTA’s inhibitory effects on T cells and enhanced antigen presentation and T cell activation [50].

Combined treatments that inhibit VISTA and other ICs have enhanced anti-tumoral responses in various cancer types [59, 63]. For example, in a murine model of colon and melanoma cancer, blocking VISTA and PD-1 together resulted in a higher survival rate and a reduction in tumor growth as compared to their monotherapy [50, 63]. On the other hand, studies show that VISTA contributes to developing resistance to immunotherapy with anti-CTLA-4 and -PD-1 in cancer patients. In melanoma patients treated with ipilimumab (PD-1 blockade), the intratumoral VISTA + lymphocytes increased, and patients with increased VISTA + TILs showed shorter disease-specific survival than the control group [65]. Gao et al. [66] showed that following ipilimumab therapy in prostate cancer patients, the expression level of VISTA and PD-L1 and infiltration of immune cells, especially CD68 + macrophages (VISTA + and PD-L1 +) that have M2 phenotype (suppressive phenotype) within the TME increased. It was shown that blocking VISTA in combination with CTLA-4 and PD-1 showed a synergistic effect and prevented the development of resistance to therapy in cancer patients [67]. Accordingly, it is suggested that inhibiting VISTA simultaneously with other ICs may not only result in more effective therapeutic outcomes but also prevent drug resistance.

The important side effect to consider in using ICIs in cancer treatment is the possibility of immune-related adverse events (irAEs), resulting in complications similar to those of autoimmune diseases caused by a disruption of self-tolerance. As a matter of fact, blocking a non-redundant NCR can result in a less suppressed immune system and a higher likelihood of over-reacted immune responses. Depending on the organ involved, these side effects include colitis, dermatitis, pneumonitis, myalgias, arthralgias, etc. [68–70]. According to a large meta-analysis, the incidence of irAE is 83% for CTLA-4 inhibitors, 72% for PD-1 inhibitors, and 60% for PD-L1 inhibitors, which are high rates [71]. In terms of the number of studies conducted on irAEs associated with VISTA, there are only a few. There was evidence that mice lacking the VISTA gene produced more IFN-γ-secreting T cells and showed chronic inflammation. Nevertheless, no organ-specific autoimmune disease developed [27]. Consequently, identifying alternative targets for ICIs, such as ligands of ICRs that suppress the immune checkpoint pathways, may increase the possibility of treating cancer patients while reducing the possibility of developing subsequent irAEs.

## Ligands of VISTA

As mentioned before, VISTA acts as both a ligand and a receptor. VISTA expressed within cells other than T cells (e.g., APCs and tumor cells) acts as a ligand via binding to unknown receptors on T cells. VISTA expressed on T cells acts as a receptor that interacts with ligands and inhibits T cells' activity via transducing downstream inhibitory pathways related to TCR [9, 13, 30]. On the other hand, VISTA shows homophilic interactions that facilitate its correlation with other VISTA proteins expressed in other cells. A confirmatory study of this issue showed that VISTA homophilic interactions intermediate macrophages’ efferocytosis and inhibition of T cell activation [14].

**Fig. 2:**
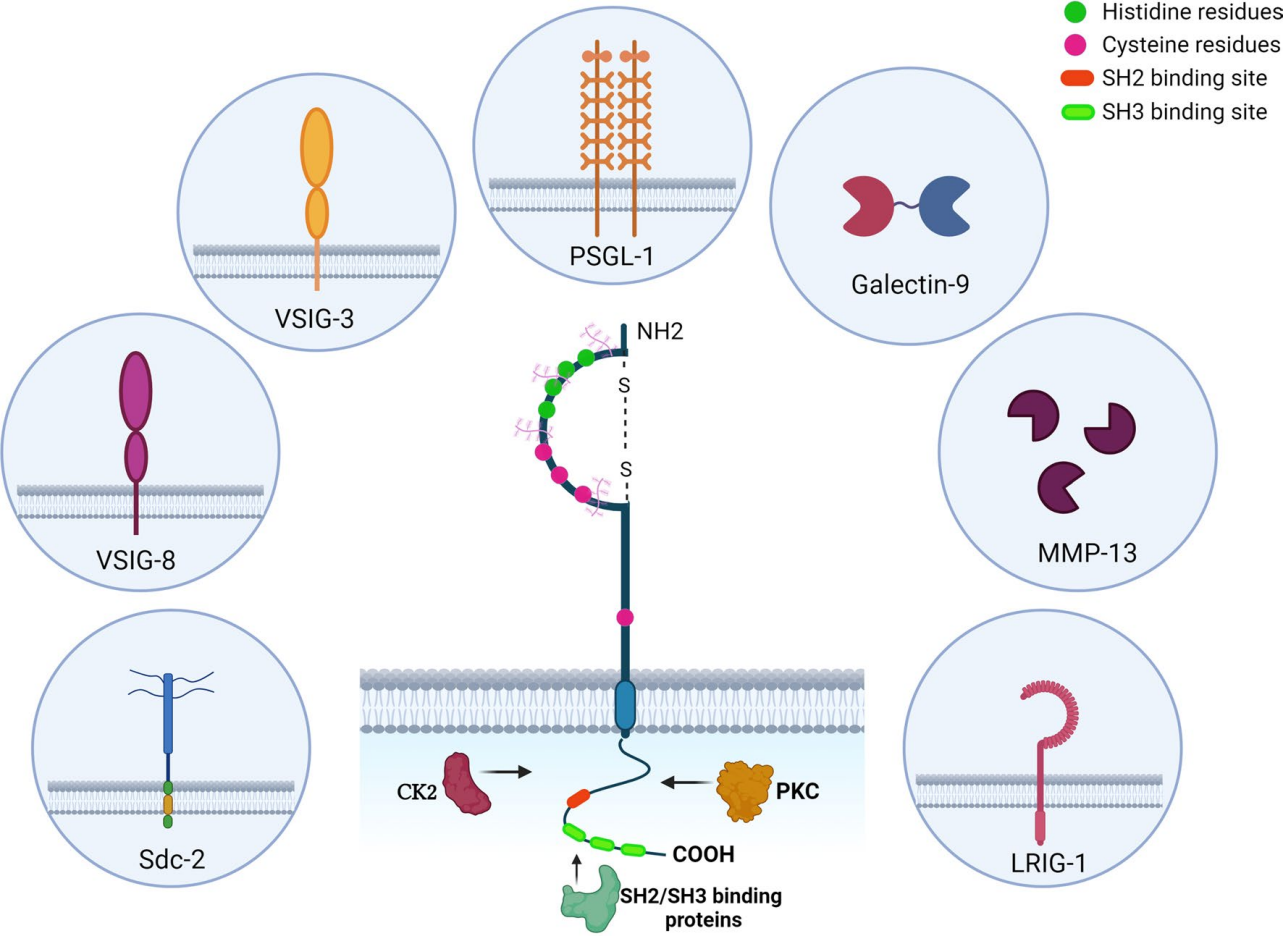
VISTA and its related ligands. VISTA interacts with its associated ligands through the ECD region, where histidine and cysteine residues play important roles. *ECD* (extracellular domain), *VISTA* (V-domain immunoglobulin suppressor of T cell activation), *PSGL-1* (P-selectin glycoprotein ligand-1), *VSIG-3* (V-Set and Immunoglobulin domain containing 3), *Gal-9* (Galectin-9), *VSIG-8* (V-Set and Immunoglobulin domain containing 8), *MMP-13* (matrix metalloproteinase-13), *Sdc2* (syndecan-2), *LRIG1* (leucine-rich repeats and immunoglobulin-like domains 1), *NH2* (N-terminus), *COOH* (C-terminus), *PKC* (phosphokinase C), *CK2* (casein kinase 2), *SH2* (Src homology domain 2), *SH3* (Src homology domain 3). ***Created with BioRender.com***

Although VISTA co-inhibitory ligands have not been fully elucidated, recent studies have suggested VSIG-3 and PSGL-1 as prominent and Galectin-9 (Gal-9), VSIG-8, matrix metalloproteinase-13 (MMP-13), syndecan-2 (Sdc2), and leucine-rich repeats and immunoglobulin-like domains 1 (LRIG1) as less well-confirmed receptors (Fig. 2).

### V-set and immunoglobulin domain containing 3 (VSIG-3)

VSIG-3, also mainly known as BT-IgSF and IGSF11, is a single-pass (type I) transmembrane protein belonging to the Ig superfamily and consists of several domains, including N-terminal (Ig-like V type and Ig-like C2 type domains), transmembrane, and C-terminal (PDZ domain). VSIG-3 gene is located on chromosome 3q13.32 and contains 431 amino acids in humans [72, 73]. Typically, VSIG-3 is present in limited tissues in the human body, including the testis and brain [72], and to a lesser extent in the ovary, adrenal, kidney, and thyroid [74]. It is interesting to note that VSIG-3 is not expressed in many vital organs of the body; hence, it was suggested to be an ideal target for immunotherapy with low side effects. VSIG-3 is an adhesion molecule and controls cell aggregation [75]. Studies have also shown its involvement in osteoclast differentiation [76], synapse formation [77], blood-testis barrier integrity [78], and the meiosis process [79]. While VSIG-3 is known to have biological functions, little is known about exactly how it acts. Regarding cancer, VSIG-3 is highly expressed in some tumor cell lines, including gastric, colon, and hepatocellular carcinoma, suggesting that VISG-3 promotes tumor cell growth and proliferation. It was demonstrated that silencing VSIG-3 reduced cell proliferation in the GC cell line, and polypeptide vaccine based on the VSIG-3 sequence stimulated the generation of VSIG3-specific cytotoxic T lymphocytes (CTLs) that killed GC cells [74]. Grelet et al. [80] showed that EMT in TME influences the expression of VSIG-3 via the lincRNA Platr18 in breast cancer models. Despite the fact that VSIG-3 has been known to play a role in cancer for a long time, there is still little information available about the role of VSIG-3 in tumorigenesis (especially in vivo) as well as its function in TME.

For the first time, Wang et al. reported that among VSIG family members, only VSIG-3 bound specifically to VISTA and did not interact with any other B7 family members. The interaction between VSIG-3 and VISTA expressed on activated human T cells was a type of adverse regulation pathway and led to a reduction in T cell proliferation, pro-inflammatory cytokine (IFN-γ, IL-2, and IL-17) and chemokine (CCL3, CCL5, and CXCL11) production, and decreased infiltration of immune cells (i.e., monocytes, DCs, tumor-associated macrophages (TAMs)) to the TME [81]. Xie et al. [73] provided the crystallographic structure of ECD and developed K284-3046 as a VSIG-3 small molecule inhibitor, which blunted the inhibitory effects of VSIG-3 on activated PBMCs. Furthermore, in a study using anti-VISTA mAb (VSTB), VISTA R54, F62, and Q63 regions were identified as interacting regions with VSIG-3 [7]. Recently, Ghouzlani et al. [82] reported significant upregulation of VSIG-3 in glioma tissues, especially in high-grade ones, compared with normal controls. They also showed a positive relation between VSIG-3 expression and other ICs, increased levels of TGF-β, and poor overall survival. Furthermore, increased expression of VSIG-3 showed an association with high immune cell infiltration (especially CD4 + and CD8 + T cells).

### P-selectin glycoprotein ligand-1 (PSGL-1)

PSGL-1 (CD162) is a disulfide-linked homodimeric type I transmembrane glycoprotein whose gene (*SELPLG*) is located on the 12q24.11 chromosome. Each subunit of PSGL-1 has three domains: extracellular, transmembrane, and cytoplasmic [83, 84]. It is widely expressed in hematopoietic cells, including lymphoid and myeloid lineage (e.g., peripheral T cells, monocytes, neutrophils, platelets, and B-cell (undetectable or very low level)), activated endothelial cells, the epithelium of the fallopian tubes, and microvascular endothelial cells. In contrast to myeloid cells, which always express the functional form of PSGL-1, CD4 + T cells only express the activated form of PSGL-1 when activated [85–87]. Lymphocyte activation regulates PSGL-1's functional binding to its ligands through post-translational modifications. Indeed, it was found that along with the activation of naïve T cells expressing significant PSGL-1, tyrosine sulfation permits PSGL-1 to interact with VISTA [88, 89].

PSGL-1 expressed in naïve T cells acts as a chemokine receptor and participates in naïve T cell homing in lymphoid tissues [90]. It also controls the proliferation and migration of neutrophils [91] and CD8 + T cells under homeostatic conditions [92]. Apart from P-selectin, PSGL-1 interacts with L-selectins and E-selectins as their ligands and acts as an adhesion molecule that mediates leukocyte trafficking and extravasation via binding to its ligands in inflamed tissues [93]. PSGL-1 engagement in DCs led to the production of IL-10, TGF-β, indoleamine 2,3-dioxygenase (IDO), and c-Fos, representing immune suppression and Treg differentiation in vitro [94]. Additionally, it inhibits hematopoietic stem cells (HSCs) proliferation in vitro [95]. PSGL-1 stimulates cytokine production in T cells, DCs, and macrophages in response to pathogens, including bacteria and viruses [96]. A study performed by Tinoco et al. [97] in *Selplg*−/−mice infected with LCMV Clone13 (as a model of chronic viral infection) indicated that PSGL-1 plays an essential role as a type of immune checkpoint protein in exhausted CD4 + and CD8 + T cells. Loss of PSLG-1 increased virus-specific CD4 + T cells, as well as improved survival and function of effector T cells compared with WT mice. Additionally, virus-specific CD8 + T cells increased survival and decreased inhibitory receptor expressions (e.g., PD-1, T-cell immunoglobulin and mucin-domain containing-3 (TIM-3), and lymphocyte-activation gene 3 (LAG3)) in *Selplg*−/−mice. Furthermore, PSGL-1 interaction with T cells suppressed TCR signaling, decreased IL-2 production, and increased inhibitory receptors, such as PD-1. Although *Selplg*−/−mice had improved T cell responses against LCMV and recovered faster than WT, the mortality rate increased due to the hyperactivation of immune responses [97]. On the basis of the points mentioned, PSGL-1 has many interactions with different stages of T cells, including activation, differentiation, and function (there is no evidence related to Treg cells) in chronic viral infection. However, the receptors through which these effects are left have not been known. On the other hand, PSGL-1 is among those immune checkpoint molecules involved in inhibiting anti-tumor responses and the migration of tumor cells [98].

PSGL-1 interacts solely with VISTA in the acidic pH range (5.85–6.5) found in the TME, where VISTA's histidine residues become deprotonated and prone to bind with PSGL-1. An in vitro study showed that blocking VISTA/PSGL-1 interaction (PSGL-1 present on human CD4 + T cells) via pH-sensitive mAb against VISTA helped to induce activation of NF-κB pathway, IFN-γ production, and proliferation of CD4 + T cells. Meanwhile, these inhibitory effects were not seen at neutral pH levels [99]. Nevertheless, much remains to be learned about how PSGL-1 affects VISTA in vivo.

### Galectin-9 (Gal-9)

Gal-9 is a member of tandem-repeat galectin proteins interacting with β-galactosid carbohydrates. Its related gene, *LGALS9*, is located on the 17q11.2 human chromosome and encodes the Gal-9 protein composed of two distinct carbohydrate-recognition domains (CRDs). It can be found in the nucleus, cytosol, plasma membrane, and ECM, and it has three isoforms in human cells, in which each isoform performs different functions and serves different purposes. The lack of a signal peptide sequence in Gal-9 suggests that it is likely to present in the exosomes and microvesicles of related cells [100, 101]. Several human tissues have shown the expression of Gal-9, especially organs and tissues of the immune system. Gal-9 provides physiological functions such as cell adhesion, differentiation (Th17 and Tregs), communication (cell signaling and host–pathogen interactions), and death (apoptosis and necrosis). It also has immunomodulatory effects and plays a crucial role in immune system functions. It is suggested as a potential therapeutic target in treating various diseases, such as RA, chronic asthma, graft rejection, pregnancy, and HIV latency, and has also been introduced as a biomarker indicating the severity of the disease, such as autoimmune disorders, atherosclerosis, infection, atopic dermatitis, chronic kidney disease, and type-2 diabetes [102]. Gal-9 expression increases not only in different types of cancer tissues [103] but also in the serum of affected patients compared to their normal counterparts [104, 105]. Notably, non-responder cancer patients to anti-PD-1 therapy exhibited higher levels of Gal-9 expression in their TILs than responders, and it was also suggested as a poor prognosis [106]. Regarding the efficacy of blocking Gal-9, in the mouse model of pancreatic ductal adenocarcinoma, Gal-9 blockade suppressed tumor progression and increased mouse survival [107]. Based on these findings, Gal-9 is suggested as an important therapeutic target and biomarker in cancer.

Many Gal-9 receptors, including protein disulfide isomerase (PDI), TIM-3, IgM, IgE, CD40, CD44, and VISTA, are predominantly expressed within immune system cells, especially T cells and mediate various activities such as TCR and BCR signaling (IgM), adaptive Treg, Th17 and osteoblast differentiation (CD44), Th2 migration (PDI), HIV infection (TIM-3), and mitigating inflammation in asthma (IgE), etc. Gal-9/PDI interaction mediates Th2 migration through ECM and increases their vulnerability to HIV infection [108]. Aside from interfering with allergic reactions by binding with IgE [109], Gal-9 also influences BCR signaling by binding to IgM on B cells [110]. The interaction between Gal-9 and CD44 facilitates the differentiation and maintenance of Treg cells [111]. As a result of binding to TIM-3, Gal-9 promotes resistance in CD4 + T cells against HIV infection, mediates immune responses to the influenza A virus [111], and induces apoptosis in TIM-3 + Th1 and Th17 cells [112]. A recent study declared the physical interaction of PD-1 with Gal-9/TIM-3 to lessen apoptosis induced by Gal-9/TIM-3 in exhausted T cells [113].

Interaction between Gal-9 and VISTA was reported for the first time using biophysical assays and immunoprecipitation studies by Yasinska et al. [10], who showed Gal-9 as a ligand interacted explicitly with VISTA with high affinity. They showed that interaction between Gal-9 produced by AML cells and VISTA (on T cells), along with TIM-3 contribution, caused the accumulation of granzyme B and increased caspase-3 activity in T cells, which finally led to the advancement of T cells toward apoptosis. The noteworthy point in this study was the absence of VISTA protein in human NK cells.

### V-set and immunoglobulin domain containing 8 (VSIG-8)

An additional member of the VSIG family, VSIG-8 (45 kDa), has also been identified as a ligand for VISTA. Its related gene is located on the 1q23.2 human chromosome and encodes; a signal sequence, an ECD domain consisting of two Ig-like V-type domains, a transmembrane domain, and a cytoplasmic domain. The human VSIG-8 protein is expressed predominantly in the spleen and CD3 + T cells of the peripheral blood as well as hair follicles, oral epithelium, and nail matrix [114–116].

In stratified epithelial cells, VSIG-8 plays a role in maintaining tight junctions [116], and its function as a co-inhibitory ligand in regulating T cells has recently been identified. Multiple experiments confirmed the specific interaction between VSIG-8 and VISTA, and the inhibitory effects of this binding on T cell activation and functions [117]. Wang et al. [118] reported that VSIG-8 binding to activated T cells significantly inhibited T cells proliferation and production of cytokines and chemokines (IFN-γ, IL-2, IL-6, IL-17, and IL-19 /MCP-1, MCP-10, and interferon gamma-induced protein 10 (IP-10/CXCL10)). It also suppressed naïve CD4 + T cell differentiation to Th1 cells.

For the first time, Molley et al. [119] reported the interaction between VSIG-8 and VISTA and suggested VSIG-8/VISTA targeting agents as a therapeutic option in cancer, autoimmune disorders, and inflammatory diseases. In addition to confirming the inhibitory effects of VSIG-8 binding to VISTA, Cheng et al. [120] developed VSIG-8 targeting small molecule compound (L557–0155), which induced PBMCs proliferation and cytokine production (TNF-α and IFN-γ). L557-0155 also inhibited tumor growth (volume and weight) and increased anti-tumor responses (tumor-specific CD8 + T cells) in the inducible melanoma model. Despite these findings, some studies have reported no evidence of binding between VISTA and VSIG-8. As mentioned, Wang et al. [81] reported no interaction between VISTA and VSIG family except VSIG-3. In another study, Georg et al. [121] developed a fusion protein containing ECD of both VSIG-8 and OX40L. ELISA binding assay and retroviral membrane display platform have not demonstrated the interaction between fusion protein and recombinant VISTA. However, this interaction was observed in human and mouse tumor cell lines expressing VISTA and macrophages. VSIG8-Fc-OX40L led to the TNF-α and IL-2 production in activated PBMCs and increased effector T cells in tumor-bearing mice.

### Matrix metalloproteinase-13 (MMP-13)

As a member of the MMPs family (zinc-dependent endopeptidases), the MMP-13 (also known as collagenase 3) protein is encoded by the gene located on the 11q22.2 human chromosome and has a crucial role in the degradation of ECM in normal physiological processes [122]. MMP-13 structure is based on four domains; signal peptide, catalytic domain, pro-domain, and hemopexin-like domain. The pro-domain, hemopexin-like, and catalytic domains are responsible for maintaining the structure of pro-MMP13, specific substrate selection, and enzyme activities, respectively [122]. Its expression is regulated via some TFs, cytokines, chemokines, hormones, growth factors and epigenetic factors [123]. The amount of MMP-13 secretion under normal conditions and its functions are regulated by low-density lipoprotein receptor-related protein 1 (LRP1) and tissue inhibitors of metalloproteinases (TIMPs), respectively. MMP-13 is mainly expressed in connective tissues and chondrocytes in humans [124]. Also, its expression has been observed in normal neuronal and epithelial cells. However, in normal conditions, there is a small amount of its term [125]. In terms of immune cells, MMP-13 expression has been observed in macrophages and DCs thus far [126, 127]. MMP-13 degrades cartilage and participates in many physiological functions, such as embryonic development, tissue remodeling, platelet aggregation and thrombosis suppression, and bone resorption. MMP-13 also is critical in pathophysiological conditions such as inflammation, atherosclerosis, RA, and cancer [128].

MMP-13 expression is increased during tumorigenesis due to disruption of the regulatory process. Its overexpression in cancer was reported in MM, breast, lung, gastric, colorectal, esophageal, prostate, thyroid, and gastric cancers, and aggressive types have shown further increases. Furthermore, MMP-13 overexpression is associated with poor prognosis, lymph node metastasis, and a shorter overall survival rate in cancer patients [123]. In a study by Howes et al. [129], the interaction of MMP-13 with platelet receptors alphaIIbbeta3 (αIIbβ3) and platelet glycoprotein (GP) VI on human platelets was shown only in solution form. Regarding VISTA and MMP-13 interaction, using pull-down and Co-IP assays showed that MMP-13, an osteoclastogenic factor secreted by MM cells, explicitly interacted with VISTA highly expressed on osteoclast cells (pre and mature) and induced osteoclast fusion and bone resorption [130]. Additionally, MMP-13 interaction with VISTA showed anti-proliferative and anti-activation effects on T cells. They declared that the VISTA/MMP-13 pathway not only regulates osteoclast function and bone resorption but also helps the immune escape of MM patients [131].

Other than the previously mentioned ligands for VISTA, Sdc2 [132] and LRIG1 [133] have been described as interacting ligands with VISTA. Rogers et al. [132] aimed to identify possible VISTA binding partners on human monocytes and found Sdc2 and heparan sulfate proteoglycan (HSPG) pathway enzymes, including HS2ST1 and B3GALT6, as binding mediators for VISTA to interact with THP-1 cells (human monocytic cell line) and effects its cellular functions. LRIG1, a transmembrane protein that negatively impacts the epidermal growth factor receptor (EGFR) signaling pathway, plays a role in tumor development when its function is disrupted. It has been shown that using anti-LRIG1 mAbs disrupted the interaction between VISTA and LRIG1 and made changes in the variety of immune cells within TME toward anti-tumoral responses. These immunological changes included increased proliferation of immune cells, increased polarization of M1 macrophages, and increased production of pro-inflammatory cytokines, especially IFN-γ [133]. Despite this, the information provided is very limited, and it is necessary to conduct further confirmatory investigations to provide additional information.

**Fig. 3:**
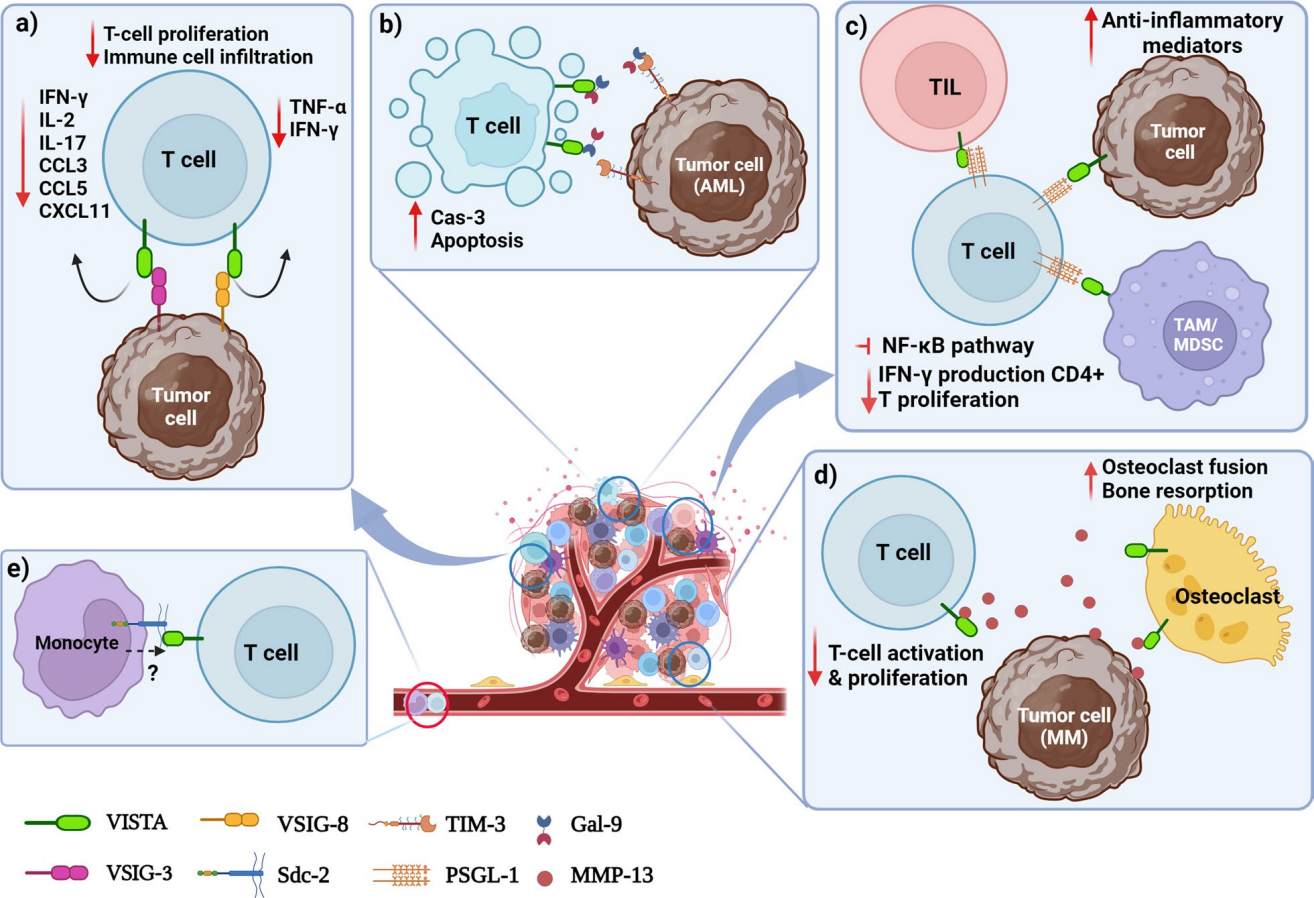
The effect of VISTA interaction with ligands and their binding effects. **a** The interaction between VSIG-3 and VSIG-8 expressed within tumor cells with VISTA on T cells causes inhibition in T cell activation and proliferation, reduction in IFN-γ, IL-2, IL-17, CCL3, CCL5, CXCL11 production, and suppression of immune cell infiltration to the TME. **b** The binding of Gal-9 secreted from the AML cells to VISTA expressed on T cells induces apoptosis in activated T cells and inhibits immune responses in the TME. **c** The interaction between PSGL-1 expressed on T cells with VISTA expressed on tumor cells, TILs, and TAMs/MDSCs not only suppresses T cell activation (blocking NF-κB pathway and reduction in IFN-γ production) and proliferation but also decreases the production of anti-inflammatory mediators in TME. **d** The MMP-13 produced by MM tumor cells binds to the VISTA expressed on the osteoclasts and T cells, causing bone resorption and T cell suppression, respectively. **e** The binding of VISTA to its unidentified ligand on monocyte surfaces is associated with Sdc-2 interactions, which have an impact on monocyte biological functions. *VISTA* (V-domain immunoglobulin suppressor of T cell activation), *VSIG-3* (V-Set and Immunoglobulin domain containing 3, *VSIG-8* (V-Set and Immunoglobulin domain containing 8), *PSGL-1* (P-selectin glycoprotein ligand-1), *Gal-9* (Galectin-9), *MMP-13* (matrix metalloproteinase-13), *Sdc2* (syndecan-2), *IFN-γ* (interferon gamma), *TNF* (tumor necrosis factor), *NF-κB* (nuclear factor kappa B), *IL* (interleukin), *CCL3* (chemokine (C–C motif) ligand 3), *CCL5* (chemokine (C–C motif) ligand 5), *CXCL11* (C-X-C motif chemokine 11), *Cas-3* (caspase 3), *TAMs* (tumor-associated macrophages), *MDSCs* (myeloid-derived suppressor cells), *TME* (tumor microenvironment), *AML* (acute myeloid leukemia), *MM* (multiple myeloma).*** Created with BioRender.com***

Detailed examinations regarding VISTA interaction with its ligands have been limited to VSIG-3 and PSGL-1, in which VSIG-3 and PSGL-1 interact with distinct but overlapping sites on VISTA's ECD and display immunosuppressive effects. In fact, this region is associated with the histidine-rich area in VISTA, including H153, H154, and H155. A key difference between these two ligands is that VISTA binds to VSIG-3 at a neutral pH (7.4) and PSGL-1 at an acidic pH (pH seen in TME) [134]. Mehta et al. [7] compared binding affinities of VSIG-3 and PSGL-1 for VISTA at different pH. They showed that at pH 7.4 (physiological pH), VISTA has a binding affinity of 20 nM for VSIG-3, while no binding was detected for PSGL-1. Conversely, at pH 6.0, while VSIG-3 showed an apparent binding affinity of 80 nM (fourfold decrease), PSGL-1 showed 4 nM (significant increase). Considering that limited information is available regarding VISTA interaction details with other ligands, we are not able to discuss more about them and the signaling pathways involved. Furthermore, it should be noted that most studies on the effects of VISTA binding to its ligands have been conducted in the context of cancer; hence, our knowledge about the impact of VISTA interaction with ligands has mostly been derived from research related to cancer, which mostly dampens anti-cancer responses and inflammation within the TME. Little is known about the effect of their interactions on the pathogenesis of autoimmune or inflammatory diseases (Fig. 3).

## VISTA and its ligands in clinical trials

As mentioned before, VISTA occupies unique features that make it stand out among others: distinct signaling pathway from CTLA-4 and PD-1/PD-L1, synergistic effects with anti-CTLA-4 and –PD-1, and involvement in resistance to anti-CTLA-4/PD-1 therapies. Therefore, targeting VISTA in TME suppresses the tumor-promoting effects and induces anti-tumoral responses. In terms of combination therapy, no information has been available regarding the simultaneous inhibition of VISTA and the use of other standard treatments, such as chemotherapy or radiotherapy. VISTA also plays a crucial role in maintaining self-tolerance, and its agonists have also been shown to be valuable in treating autoimmune and inflammatory diseases. According to the most recent multiple preclinical and clinical studies, VISTA appears to have tremendous therapeutic potential, either as an agonist or antagonist (Table 1).

**Table 1:** Current ongoing VISTA therapeutics (small molecule inhibitors and mAbs) in preclinical and clinical development

Intervention	Description	Target	Cancer type	Developed by	Phase	Identifiers	Location	Status
Antibodies (Agonist)								
4C11	mAb	VISTA	Asthma	Yale University	Preclinical	–	–	–
7G1	mAb	VISTA	Psoriasis	University of Toronto	Preclinical	–	–	–
Small molecule inhibitors								
AUPM-493	small molecule	VISTA & VSIG-8	Melanoma, and colon cancer	Aurigene, Inc	Preclinical	–	–	–
CA-170	small molecules	VISTA & PD-L1/L2	Advanced Solid Tumors or Lymphomas	Curis, Inc	Phase I	NCT02812875	U.S.A	Completed
CA-170	small molecules	VISTA & PD-L1/L2	Lung cancer, Hodgkin lymphoma, head and neck/oral cavity cancer, and MSI-high cancer	Aurigene & Curis, Inc	Phase II	CTRI/2017/12/011026	India	Terminated
Blocking antibodies (Antagonist)								
PMC-309	IgG1 mAb	VISTA	In vitro assays and multiple syngeneic mouse tumor models	PharmAbcine, Inc	Preclinical	–	–	–
KVA 12.1	IgG1 mAb	VISTA	In vitro assays and multiple syngeneic mouse tumor models	Kineta, Inc	Preclinical	–	–	–
APX-201	mAb	VISTA	In vitro assays and multiple syngeneic mouse tumor models	Apexigen, Inc	Preclinical	–	–	–
VTX-0811	mAb	PSGL-1	In vitro assays and multiple syngeneic mouse tumor models	Verseua, Inc	Preclinical	–	–	–
SNS-101	IgG1 mAb	VISTA/PSGL-1	In vitro assays and multiple syngeneic mouse tumor models	Sensei Bio, Inc	Preclinical	–	–	–
IMT-18	mAb	VSIG-3	In vitro assays and multiple syngeneic mouse tumor models	iOMx, Inc	Preclinical	–	–	–
BMS767	mAb	VISTA	In vitro assays and multiple syngeneic mouse tumor models	Bristol-Myers Squibb, Inc	Preclinical	–	–	–
IGN-381	mAb	VISTA	Hematological cancer and Solid tumors	Igenica Biotherapeutics, Inc	Preclinical	–	–	–
SG7	mAb	VISTA/PSGL-1 & VSIG-3	In vitro assays and multiple syngeneic mouse tumor models	Mehta et al	Preclinical	–	–	–
VSTB112 (JNJ-61610588)	IgG1 mAb	VISTA	Lung, pancreatic, head and neck, colorectal, and cervical cancers	ImmuNext/Janssen, Inc	Phase I	NCT02671955	U.S.A	Terminated
CI-8993 (Onvatilima)	IgG1 mAb	VISTA	Advanced solid tumors	Curis, Inc	Phase I	NCT04475523	U.S.A	Recruiting
W018	mAb + Pembrolizumab	VISTA/PSGL-1	Locally advanced or metastatic solid tumors	Pierre Fabre Medicament, Inc	Phase I	NCT04564417	France&Spain	Recruiting
HMBD-002	IgG4 mAb	VISTA/VSIG-3 & LRIG1	Advanced solid tumors	Hummingbird Bioscience, Inc	Phase I/II	NCT05082610	U.S.A	Recruiting

### Agonists

VISTA agonistic mAbs promote the activity of VISTA; induction of activation-induced cell death (AICD) and enhancement of peripheral T cell tolerance, inhibit myeloid chemotaxis, and reprogramming macrophages towards an anti-inflammatory profile [16, 135]. Hence, they can be a suitable treatment option for inflammatory and autoimmune disorders. Recent animal studies showed that using VISTA agonist antibodies results in immunomodulatory effects, such as suppressing NF-κB signaling pathway and production of pro-inflammatory cytokines [16]. In GVHD, targeting VISTA in donor CD4 + T cells with agonistic antibody before the transfer led to the deletion of donor alloreactive T cells via the T cell-intrinsic pathway and prevented disease [30]. As mentioned before, the VISTA agonist antibody (4C11) suppressed lung inflammation and reduced the severity of the disease [41]. Five agonistic anti-VISTA mAbs are under investigation in autoimmune disorders, 8G8, INX803, 7G1, 7G5, and 7E12, which target VISTA in mice, humans, or both. Studies indicate that these antibodies induce VISTA signaling and suppress immunity, although little information about their effects is available [136, 137]. It should be noted that only VISTA is currently being developed as a checkpoint agonist in clinical studies.

### Antagonists

#### Small molecule inhibitors

##### CA-170

A small molecule inhibitor named CA-170, taken orally, targets VISTA (H strand) and PD-L1/L2 pathways without interrupting PD-1/PD-L1 interaction. Since 2015, Curis has licensed the technology from Aurigene, and in vitro studies showed that CA-170 promotes cell proliferation and IFN-γ production in T cells suppressed by VISTA or PD-1/PD-L1 [138, 139]. In syngeneic mouse models of melanoma and colon cancer (B16, CT26, and MC38), CA-170 suppressed tumor growth, promoted the activation of peripheral T cells, and activation of TILs [140]. Phase I trial (NCT02812875) showed its safety and effectiveness in solid tumors and lymphoma. Patients involved presented increased activated CD4 + and CD8 + T cells in the periphery [141]. Phase II studies in lung cancer, Hodgkin lymphoma, head and neck/oral cavity, and MSI-high cancers are currently underway by Aurigene in India [142]. Despite all these findings, the binding of human VISTA to CA-170 was not confirmed [143].

##### AUPM-493

Small molecule developed by Aurigene and acts as PD-L1 and VISTA antagonist. In a preclinical study, AUPM-493 suppressed the interaction between VISTA and VSIG-8, which led to the activation of T cells and IFN-γ production. It also showed anti-tumoral effects in syngeneic models of melanoma and colon cancer [117].

#### Blocking antibodies

##### VSTB112

Regarding mAbs blocking VISTA, JNJ-61610588 or VSTB112 is the first humanized IgG1κ antibody developed by ImmuNext/Janssen, targeting human VISTA through C–C′ loops (H121 and H122 residues) and adjacent Helix. There is no pH dependence on the interaction between VSTB and VISTA [144]. It was found that VSTB112 suppressed VISTA signaling in vitro and also tumor regression in a mouse model of bladder cancer (human VISTA knock-in mice) as a result [145]. In 2016, Janssen Biotech started a phase I trial (NCT02671955) to assess its safety, tolerability, and pharmacokinetics in advanced tumors, including lung, pancreatic, head and neck, colorectal, and cervical cancers [146]. The study was prematurely terminated after 2 years for unknown reasons in a situation where one of 12 patients experienced cytokine release syndrome.

##### CI-8993

Curis is currently pursuing VSTB112 (JNJ-61610588) as CI-8993** (**Onvatilimab) in the phase I trial (NCT04475523) in relapsed/refractory solid tumors [147]. It is important to note that even at subtherapeutic doses, CI-8993 triggers a significant release of cytokines that can cause neurotoxicity, maybe due to its cell-depleting IgG1 backbone.

##### W018 (K01401-020)

The newest anti-VISTA mAb with IgG1 subtype, developed by Pierre Fabre, inhibits PSGL-1 binding to VISTA at pH 6–7.4 [148]. W018 phase I trial (NCT04564417) has recently commenced (149).

##### BMS767 (P1-068767)

Anti-VISTA human mAb investigated by Bristol-Myers Squibb. It is the only VISTA pH-sensitive antibody interacting with VISTA (H121, H122, and some C–C' loop residues) at pH 6.0 (not physiological pH) [144].

##### SG7

Developed by yeast surface display technology and has inhibitory effects against VISTA. It has overlapping epitopes with VSTB112 and BMS767, binding only to human VISTA. However, it has unique regions for binding to VISTA expressed in murine and cynomolgus monkey, which makes SG7 species cross-reactive antibody with high affinity. Jurkat T cell activation assay showed that the activation level of T cells was restored by SG7, and blocking VISTA via SG7 reduced tumor growth in syngeneic tumor models. SG7 was also found to reduce the number of polymorphonuclear MDSCs (PMN-MDSCs) in a TME from 4T1-bearing mice and increased the number of CD4 + and CD8 + T cells. PMN-MDSCs are cells with a high expression of VISTA and suppress anti-tumoral responses in TME. Nevertheless, no effect was observed on other examined myeloid cells such as CD11c + DCs, CD11b + macrophages, and monocytic myeloid-derived suppressor cells (M-MDSCs). SG7 inhibited VISTA's interaction with VSIG-3 at pH 7.4 and PSGL-1 at pH 6.0, mainly via H122 and E125 residues [144].

##### HMBD-002

An IgG4 anti-VISTA mAb developed by Hummingbird Biosciences, which primarily interacts with the C–C' loop of VISTA, where VISTA interacts with VSIG-3 and LRIG1. It neutralizes VISTA functions without depleting VISTA + cells by acting via an Fc-independent mechanism. Regarding VISTA/VSIG-3 interactions, HMBD-002 resolved the suppressory effects of VSIG-3, and anti-CD3-activated T cells produced IFN-γ. Additionally, it has been shown that HMBD-002 reverses the inhibitory effects of MDSCs on T cells, suppresses tumor cell invasion, and, most importantly, enables T cells to shift toward Th1/Th17 [150]. In several humanized and syngeneic murine models of breast, colorectal, and lung cancer, HMBD-002 showed therapeutical effects and suppressed tumor growth without apparent toxicity. A preclinical study showed that in combination with pembrolizumab (anti-PD-L1), HMBD-002 demonstrated superior efficacy, particularly in tumors with high infiltration of MDSCs [151–153]. It is now under investigation in the phase 1/2 trial (NCT05082610) as a single agent and combined with pembrolizumab in advanced solid tumors expressing VISTA [154].

Both CI-8993 and W018 induce anti-tumor effects through Fc-dependent activities of their IgG1, which frequently result in ADCC or complement-dependent cytotoxicity (CDC)-mediated cell death [155]. It is important to note that VISTA is expressed in a wide range of healthy cells, which means that this activity can cause the death of a large number of cells that are not targeted. HMBD-002 epitope is distinct from CI-8993 and W018 and exhibits high binding specificity for VISTA in various species (human, rat, monkey, and murine orthologs). VSTB112, SG7, and BMS767 all interact with the H122 residue within VISTA to effectively inhibit the binding of both VSIG-3 and PSGL-1. SG7 showed more affinity binding around 25 to 50 fold compared with VSTB112 or BMS767. While the interaction between BMS767 and VISTA is pH-dependent, the binding of SG7 and VSTB112 is not, which makes BMS767 a potential anti-VISTA targeting mAb homing TME. Furthermore, VSTB112 and BMS767 show depletion in VISTA-expressing cells (active Fc), but SG7 does not (dead Fc) [144, 156]. Currently, some mAbs are in the preclinical stage of development, such as KVA 12.1 [157], PMC-309 [158], APX-201, VTX-0811, SNS-101 [159], IMT-18, and IGN-381 for which only a few publications have been published so far, and some are still in the development process [160, 161].

## Conclusions and future perspectives

There is no doubt that VISTA has attracted attention in immunotherapy thanks to its distinguishing features compared with other NCRs, where it shows more profound immunoregulatory effects as a result of these options. The impressive results that have been published from treatments based on VISTA targeting highlight the importance and highest value of examining VISTA in more detail [67, 135]. Regarding therapy efficacy, VISTA agonists appear to be more effective in autoimmune and inflammatory disorders than antagonists in cancer because of VISTA-associated irAEs and bi-directional role. However, it should be kept in mind that in MS and lupus cases, various elements such as genetics and inflammatory factors could affect the expression of VISTA. In this regard, investigating the expression pattern of VISTA in autoimmune disorders may be helpful before starting any therapy based on this gene.

Based on the information discussed above, it is clear that all VISTA ligands, apart from VISTA, play an important role in tumor development and growth. In some cases, their importance is so great that they have even been proposed as targets for immunotherapy. Among the VISTA ligands, PSGL-1 and VSIG-3 are valuable options to be considered due to their unique expression and functions, which may reduce side effects related to VISTA targeting. Nevertheless, newly identified ligands, such as Gal-9 and MMP-13, can also be investigated as potential targets blocking the VISTA pathway in the future. Because blocking their interaction with VISTA showed that their inhibition has the potential not only to activate immune responses but also suppress tumor growth. However, in proposing VISTA ligands as alternative targets for blocking the VISTA inhibitory pathway, consideration should be given to their functions in other parts of the body as well as the immune system. Because even if its inhibition suppresses tumor cell growth, it can result in serious secondary complications. Therefore, in determining which of the identified ligands for VISTA should be targeted, choosing the most effective option in suppressing VISTA signaling with fewer irAEs is advisable.

Regarding designing mAbs, it would be advantageous to choose specific residues shared between multiple VISTA ligands to inhibit all relevant VISTA pathways and/or the choice of non-overlapping regions to block a particular path. This case requires identifying the details of the regions through which the ligands are attached to VISTA. Moreover, the Fc activity of the antibodies is another critical factor to consider. Fc-independent antibodies have a high priority in the effort to eliminate ADCC and CDC. It is also possible for bispecific antibodies to be used as a combination therapy in order to inhibit VISTA and its related pathways, as well as other ICs.

On the other hand, environmental factors are also important for improving therapy efficacy. As discussed before, the environment’s pH is a critical factor in VISTA’s performance and binding to its ligands [162]. Hence, some strategies could be used to optimize this factor. For example, PSGL-1 interacts with VISTA in an acidic pH environment and, therefore, has the highest priority to inhibit the VISTA pathway in TME and would be a suitable therapeutic option for cancer therapy. BMS767 is the only VISTA-targeting antibody developed based on this point.

Altogether, it is clear that VISTA's ligands are just as important as VISTA itself, and their interactions play a significant role in many diseases related to the immune system, especially cancer. Therefore, VISTA and its ligands can be quite promising candidates when it comes to considering new immunotherapeutic targets. Nevertheless, VISTA's interaction with ligands, especially other than PSGL-1 and VSIG-3, their related effects, and intracellular pathways remain a work in progress. As a result, it is imperative that more studies be conducted in vitro, particularly in vivo, to fill these knowledge gaps and to maximize the potential of targeting VISTA through new therapeutic approaches.

## Data Availability

Not applicable.

## Abbreviations

VISTA: V-domain immunoglobulin suppressor of T cell activation
ICs: Immune checkpoints
ICRs: Immune checkpoint receptors
CTLA-4: Cytotoxic T-lymphocyte-associated protein 4
PD-1: Programmed cell death protein 1/PD-L1 (Programmed death-ligand 1)
FDA: Food and drug administration
NSCLC: Non-small cell lung cancer
PD-1H: PDCD-1 homolog
SISP1: Stress-induced secreted protein 1
DD1a: Death domain1alpha
Dies1: Differentiation of embryonic stem cells 1
ITAM: Immunoreceptor tyrosine-based activation motif
ITIM: Immunoreceptor tyrosine-based inhibitory motif
ITSM: Immunoreceptor tyrosine-based switch motif
SH3: Src homology domain 3
SH2: Src homology domain 2
PKC: Phosphokinase C
CK2: Casein kinase 2
ECD: Extracellular domain
TME: Tumor microenvironment
EAE: Experimental autoimmune encephalomyelitis
MS: Multiple sclerosis
Th2: T helper 2
MDSC: Myeloid-derived suppressor cells
MAIDS: Murine model of AIDS
mAb: Monoclonal antibody
TNF: Tumor necrosis factor
IL: Interleukin
CXCL2: C-X-C motif chemokine ligand 2
DCs: Dendritic cells
TLRs: Toll-like receptors
MyD88: Myeloid differentiation primary response 88
APCs: Antigen-presenting cells
CNS: Central nervous system
CKO: Conditional knockout
BM: Bone marrow
PBMCs: Peripheral blood mononuclear cells
FoxP3 +: Forkhead box P3
Tregs: Regulatory T cells
NK: Natural killer
TFs: Transcription factors
NF-κB: Nuclear factor kappa B
HIF-1α: Hypoxia-inducible factor 1-alpha
FOXD3: Forkhead box D3
miRNA: MicroRNA
LPS: Lipopolysaccharide
IFN-γ: Interferon-γ
Tfh: T follicular helper
DLE: Discoid lupus erythematosus
SLE: Systemic lupus erythematosus
RRMS: Relapsing–remitting multiple sclerosis
RA: Rheumatoid arthritis
GVHD: Graft-versus-host disease
MCP-1: Monocyte chemoattractant protein-1
WT: Wild-type
TILs: Tumor-infiltrating lymphocytes
GC: Gastric cancer
AML: Acute myeloid leukemia
OSCC: Oral squamous cell carcinoma
IrAEs: Immune-related adverse events
TGF-β: Transforming growth factor beta
VSIG-3: V-Set and Immunoglobulin domain containing 3
PSGL-1: P-selectin glycoprotein ligand-1
Gal-9: Galectin-9
VSIG-8: V-Set and Immunoglobulin domain containing 8
MMP-13: Matrix metalloproteinase-13
Sdc2: Syndecan-2
LRIG1: Leucine-rich repeats and immunoglobulin-like domains 1
CTLs: Cytotoxic T lymphocytes
BC: Breast cancer
IDO: Indoleamine 2,3-dioxygenase
HSCs: Hematopoietic stem cells
TIM-3: T-cell immunoglobulin and mucin-domain containing-3
LAG3: Lymphocyte-activation gene 3
CRDs: Carbohydrate-recognition domains
MM: Multiple myeloma
PDI: Protein disulfide isomerase
LRP1: Lipoprotein receptor-related protein 1
TIMPs: Inhibitors of metalloproteinases
HSPG: Heparan sulfate proteoglycan
EGFR: Epidermal growth factor receptor
AICD: Activation-induced cell death
CDC: Complement-dependent cytotoxicity
PMN-MDSCs: Polymorphonuclear MDSCs
M-MDSCs: Monocytic myeloid-derived suppressor cells
